# Compensatory mechanisms amidst demyelinating disorders: insights into cognitive preservation

**DOI:** 10.1093/braincomms/fcae353

**Published:** 2024-11-12

**Authors:** Noor Z Al Dahhan, Julie Tseng, Cynthia de Medeiros, Sridar Narayanan, Douglas L Arnold, Brian C Coe, Douglas P Munoz, E Ann Yeh, Donald J Mabbott

**Affiliations:** Neurosciences and Mental Health Program, Research Institute, The Hospital for Sick Children, Toronto, Ontario, M5G 0A4, Canada; McGovern Institute for Brain Research, Massachusetts Institute of Technology, Cambridge, MA, 02139, USA; Neurosciences and Mental Health Program, Research Institute, The Hospital for Sick Children, Toronto, Ontario, M5G 0A4, Canada; Neurosciences and Mental Health Program, Research Institute, The Hospital for Sick Children, Toronto, Ontario, M5G 0A4, Canada; Department of Neurology and Neurosurgery, Montreal Neurological Institute and Hospital, McGill University, Montreal, H3A 2B4, Canada; Department of Neurology and Neurosurgery, Montreal Neurological Institute and Hospital, McGill University, Montreal, H3A 2B4, Canada; Centre for Neuroscience Studies, Queen’s University, Kingston, Ontario, K7L 3N6, Canada; Centre for Neuroscience Studies, Queen’s University, Kingston, Ontario, K7L 3N6, Canada; Neurosciences and Mental Health Program, Research Institute, The Hospital for Sick Children, Toronto, Ontario, M5G 0A4, Canada; Department of Neurology, The Hospital for Sick Children, Toronto, Ontario, M5G 1X8, Canada; Department of Paediatrics, University of Toronto, Toronto, Ontario, M5G 1X8, Canada; Neurosciences and Mental Health Program, Research Institute, The Hospital for Sick Children, Toronto, Ontario, M5G 0A4, Canada; Department of Psychology, University of Toronto, Toronto, Ontario, M5S 3G3, Canada

**Keywords:** demyelinating disorders, structure-function coupling, network connectivity, cognitive performance, compensatory mechanisms

## Abstract

Demyelination disrupts the transmission of electrical signals in the brain and affects neurodevelopment in children with disorders such as multiple sclerosis and myelin oligodendrocyte glycoprotein-associated disorders. Although cognitive impairments are prevalent in these conditions, some children maintain cognitive function despite substantial structural injury. These findings raise an important question: in addition to the degenerative process, do compensatory neural mechanisms exist to mitigate the effects of myelin loss? We propose that a multi-dimensional approach integrating multiple neuroimaging modalities, including diffusion tensor imaging, magnetoencephalography and eye-tracking, is key to investigating this question. We examine the structural and functional connectivity of the default mode and executive control networks due to their significant roles in supporting higher-order cognitive processes. As cognitive proxies, we examine saccade reaction times and direction errors during an interleaved pro- (eye movement towards a target) and anti-saccade (eye movement away from a target) task. 28 typically developing children, 18 children with multiple sclerosis and 14 children with myelin oligodendrocyte glycoprotein-associated disorders between 5 and 18.9 years old were scanned at the Hospital for Sick Children. Tractography of diffusion MRI data examined structural connectivity. Intracellular and extracellular microstructural parameters were extracted using a white matter tract integrity model to provide specific inferences on myelin and axon structure. Magnetoencephalography scanning was conducted to examine functional connectivity. Within groups, participants had longer saccade reaction times and greater direction errors on the anti- versus pro-saccade task; there were no group differences on either task. Despite similar behavioural performance, children with demyelinating disorders had significant structural compromise and lower bilateral high gamma, higher left-hemisphere theta and higher right-hemisphere alpha synchrony relative to typically developing children. Children diagnosed with multiple sclerosis had greater structural compromise relative to children with myelin oligodendrocyte glycoprotein-associated disorders; there were no group differences in neural synchrony. For both patient groups, increased disease disability predicted greater structural compromise, which predicted longer saccade reaction times and greater direction errors on both tasks. Structural compromise also predicted increased functional connectivity, highlighting potential adaptive functional reorganisation in response to structural compromise. In turn, increased functional connectivity predicted faster saccade reaction times and fewer direction errors. These findings suggest that increased functional connectivity, indicated by increased alpha and theta synchrony, may be necessary to compensate for structural compromise and preserve cognitive abilities. Further understanding these compensatory neural mechanisms could pave the way for the development of targeted therapeutic interventions aimed at enhancing these mechanisms, ultimately improving cognitive outcomes for affected individuals.

## Teaser

Demyelination disrupts brain signal transmission and neurodevelopment in children with demyelinating disorders, often leading to cognitive impairments. However, some children maintain cognitive function despite structural injury. Al Dahhan *et al*. integrate neuroimaging modalities to examine compensatory neural mechanisms, suggesting increased functional connectivity compensates for structural compromise and preserves cognitive abilities.

## Graphical Abstract

**Graphical Abstract:**
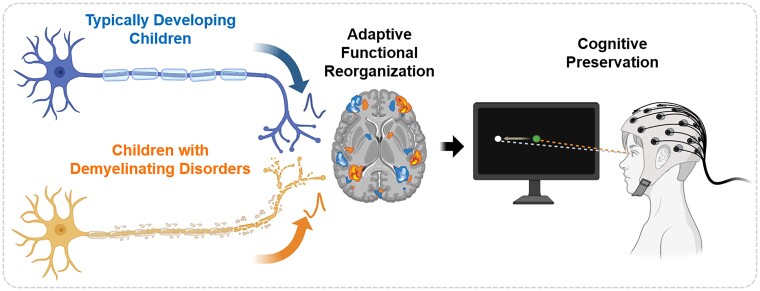

## Introduction

Demyelination, characterized by the loss or damage of the protective myelin sheath surrounding nerve fibres, disrupts the transmission of electrical signals in the brain.^1-7^ This disruption can profoundly impact the developing brain^8-13^ and contribute to neurodevelopmental challenges in children with demyelinating disorders, such as multiple sclerosis (MS) and myelin oligodendrocyte glycoprotein-associated disorders (MOGAD).^14-17^ In a third of cases of children with demyelinating disorders, the influence of demyelination on the developing brain negatively impacts cognitive functioning.^14-17^ The cognitive domains most frequently affected in children with demyelinating disorders include difficulties with attention, memory and information processing speed.^11-13,15,18-24^ These cognitive domains are proposed to be impacted by the structural and conductive properties of white matter through its influence on functional network dynamics.^2,9,25-29^ Subsequently, cognitive performance is hypothesized to be sub-served by coherently oscillating neuronal populations that produce temporal windows for communication.^2-7^ In addition to white matter injury, gray matter demyelination is also implicated in cognitive impairments in children with demyelinating disorders.^18,30,31^

While cognitive impairments are common in these conditions, some children exhibit preserved cognitive function despite significant white matter injury.^20,32,33^ These findings raise an important question: alongside the degenerative process, do compensatory neural mechanisms exist to mitigate the effects of myelin loss? These compensatory mechanisms refer to the brain’s ability to adapt and reorganize in response to injury or damage to maintain functionality, potentially aiding in the preservation of cognitive function.^34-36^ Investigating this dynamic interplay between degeneration and potential compensation is crucial for furthering our understanding of the pathophysiology of MS and MOGAD-related disorders.

**Figure 1:**
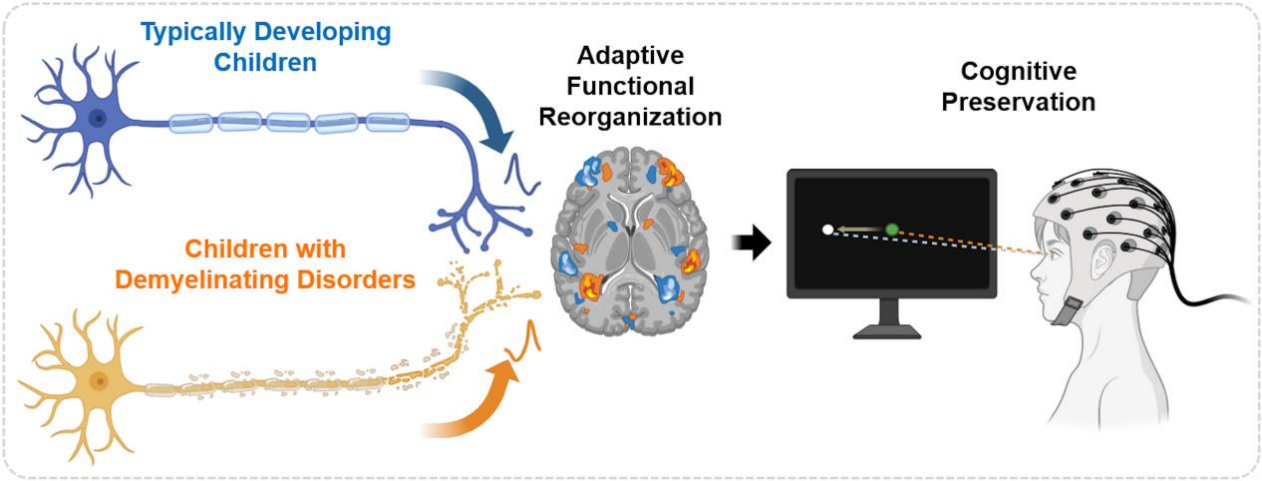
**Compensatory mechanisms amidst demyelinating disorders, leading to cognitive preservation.** Despite the progressive white matter microstructural changes experienced by children with demyelinating disorders, such as MS and MOGAD, many of these children do not differ from typically developing children on cognitive outcomes, such as saccade reaction times and direction errors on pro- and anti-saccade tasks. This cognitive preservation is hypothesized to be due to an adaptive functional reorganisation in response to white matter changes, such as an increased recruitment of parallel pathways as a compensatory mechanism. Examining how network neural function adapts and compensates for structural compromise is important for not only understanding the neural basis of typical cognition, but also for understanding how network disturbances may be associated with cognitive impairments.

We hypothesize that this cognitive preservation may stem from an adaptive functional reorganisation in response to white matter changes, such as an increased recruitment of parallel pathways (Fig. 1).^34,37,38^ To test this hypothesis and evaluate cognitive function in children with demyelinating disorders, it is key to grasp how demyelination affect both neural function and behaviour. We propose that a multi-dimensional approach integrating multiple neuroimaging modalities, including diffusion tensor imaging (DTI), magnetoencephalography (MEG) and eye-tracking, is key to investigating this hypothesis. Specifically, DTI and MEG are paramount for exploring brain structure and function.^28^ While DTI facilitates the examination of white matter tracts,^39-42^ MEG has the capability to pinpoint temporal correlations in spatially remote neural regions.^43-45^ Intracellular and extracellular microstructural parameters can also be extracted using a white matter tract integrity (WMTI) model to specific inferences on myelin and axon structure.^46-49^ These two neuroimaging modalities, provide a holistic perspective on brain connectivity and dynamics, which is essential for probing neurological conditions and cognitive processes.^28^

**Figure 2:**
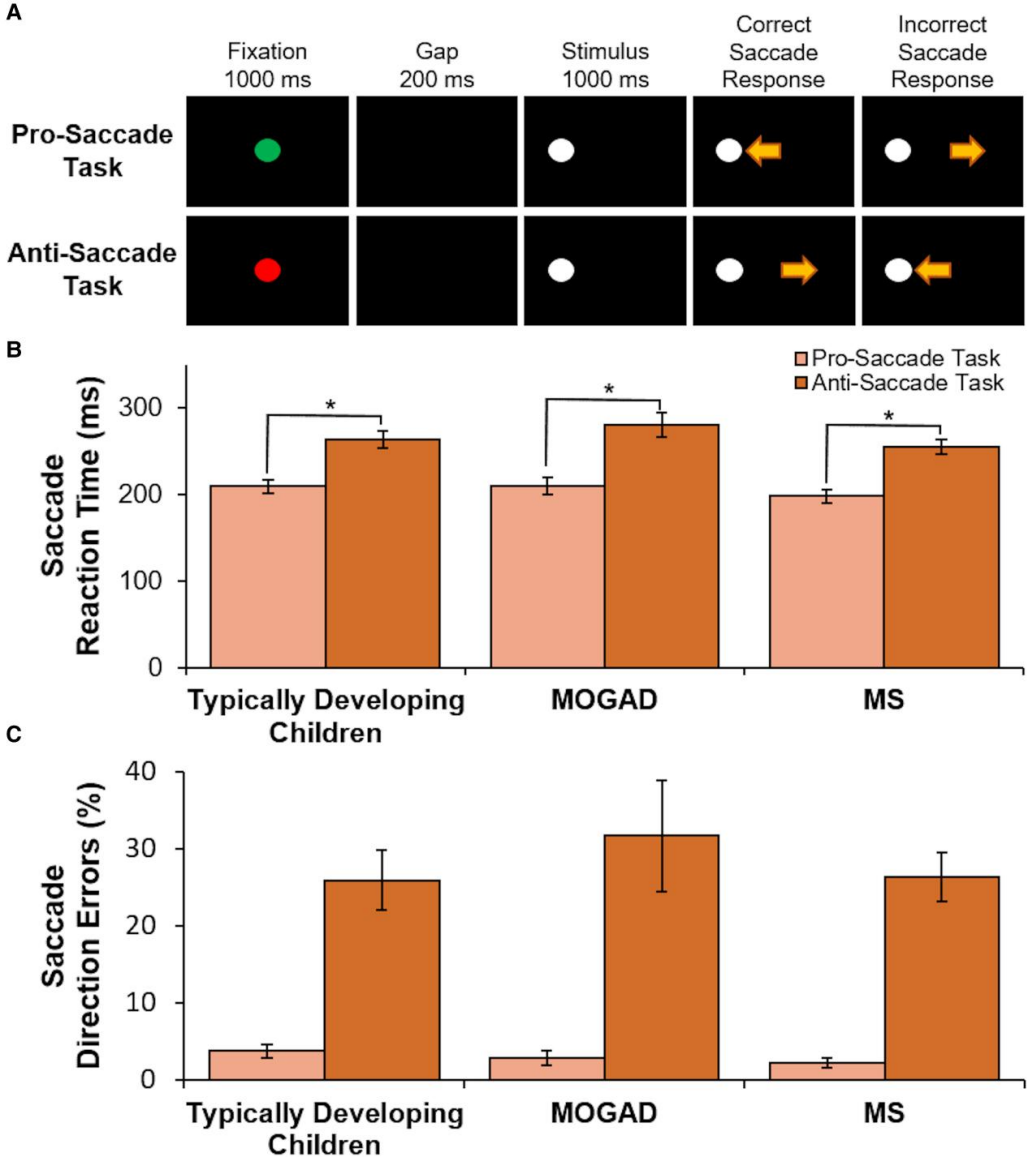
**Similar behavioural group performance on (A) the pro- and anti-saccade tasks between (B) typically developing children and children with demyelinating disorders on saccade reaction times and (C) saccade direction errors.** During the pro- and anti-saccade task (**A**), trials began with a central instructional cue on a black background for 1000 ms; a green fixation cue indicated a pro-saccade trial, and a red fixation cue indicated an anti-saccade trial. After a 200 ms gap period, a white peripheral stimulus appeared 10° to the left or right of the central instructional cue for 1000 ms. Participants were instructed to look towards the white peripheral stimulus during pro-saccade trials, and away from the stimulus in the opposite direction during anti-saccade trials. Both trial conditions and stimulus locations were pseudo-randomly interleaved. Saccade reaction time was defined as the time from stimulus appearance to the first saccade away from fixation that exceeded 30°/s. Two-sample *t*-tests examined group differences on pro- and anti-saccade reaction times and direction errors for 28 typically developing children, 18 children with MS and 14 children with MOGAD. Within groups (**B**), participants had longer saccade reaction times and (**C**) greater direction errors on the anti- versus pro-saccade task (*P* < 0.05); however, there were no behavioural group differences on either task (*P* > 0.05). *Note.* Standard errors are shown; * = *P* < 0.05; MOGAD, children diagnosed with myelin oligodendrocyte glycoprotein-associated disorders; MS, children diagnosed with multiple sclerosis.

In addition, conducting MEG scanning while simultaneously recording eye movements can inform our understanding of neural activity and its relationship to cognitive processes in children with demyelinating disorders. Moreover, evaluating functional metrics in children with demyelinating disorders is vital for prognosis and clinical decision-making. However, existing assessment tools, such as cognitive or visual testing, are often time-consuming and require specialized training. Thus, there is a critical need for a user-friendly screening tool that can offer clinically relevant functional insights in this population. Eye-tracking presents a promising and feasible solution as it is low-cost, non-invasive and easy to administer.^50^ With its well-characterized circuitry, the oculomotor system offers a valuable window into cognitive processes. For example, tasks such as the pro- (eye movement towards target) and anti-saccade (eye movement away from target) tasks engage the oculomotor system, and have been shown to uncover potential deterioration linked to demyelinating disorders (Fig. 2A).^50,51^ Specifically, assessing saccade performance during the pro- and anti-saccade tasks is a valuable tool for probing executive functions, particularly inhibitory control, working memory and cognitive flexibility.^50,52,53^ The distinction between these tasks lies in their emphasis. The pro-saccade tasks isolate visual and motor aspects.^52,54^ The anti-saccade task assesses inhibitory control as participants are required to suppress the automatic response (pro-saccade) to a visual target and generate a voluntary, controlled eye movement in the opposite direction.^52,54^ Saccade reaction times during anti-saccade trials have been shown to be longer relative to pro-saccade trial, and this delay has been associated with the top–down inhibition of a pro-saccade during anti-saccade trials.^50,54-56^ Quantitative assessment of oculomotor performance can thus serve as a vital tool for tracking disease disability, disease progression, treatment effectiveness and functional recovery in children with demyelinating disorders.^52,54^

By examining the interaction among oculomotor behaviour, neural activity and white matter organisation, we aim to examine the brain's adaptive and compensatory responses to myelin loss. Executive functions, particularly inhibitory control, working memory and cognitive flexibility, assessed by the pro- and anti-saccade tasks are driven by the speed of neuronal transmissions among multiple brain networks, including the default mode (DMN) and executive control (ECN) networks.^57-60^ Thus, we specifically focused on examining the structural and functional connectivity of these two networks due to their significant roles in supporting higher-order cognitive processes.^57^ However, the precise mechanisms underlying adaptive functional reorganisation in response to structural compromise of the DMN and ECN remain unknown, yet investigating these processes holds promise for understanding preserved cognition in children with demyelinating disorders. We predict that structural connectivity is associated with saccade reaction times and direction error rates on the pro- and anti-saccade tasks directly and indirectly via neural synchrony. This multi-dimensional approach contributes to our understanding of how disease disability and degeneration affect brain network connectivity, and holds implications for therapeutic interventions aimed at enhancing compensatory neural mechanisms to improve cognitive outcomes for affected individuals.

## Materials and methods

### Participants

32 children with demyelinating disorders [MS (*n* = 18) and anti-MOG antibody (ab) positive individuals with recurrent disease (*n* = 14)] were recruited from the Neuroinflammatory Disorders Program at the Hospital for Sick Children (SickKids; Toronto, ON). 28 age-matched typically developing children were recruited from the community.

MS was diagnosed based on the Revised 2017 McDonald criteria.^61^ Children with anti-MOG-ab positive disease met these criteria: (i) onset before 18 years of age, (ii) MOG-ab positivity confirmed by a live cell-based assay at least 3 months after onset; and (iii) recurrence of neuroinflammation more than 12 weeks after the first episode. MOG-ab testing was conducted through a commercial laboratory at Oxford University using live cell-based methods.^62^ All children were retrospectively evaluated using MOGAD diagnostic criteria and met these criteria.^63^ Children with demyelinating disorders were excluded if they had a history of other neurological conditions, major medical or psychiatric comorbidities, learning disabilities, traumatic brain injury, substance abuse, non-demyelinating causes of white matter dysfunction, cerebral palsy or had been treated with steroids within the past 6 months. Additional inclusion criteria for children with demyelinating disorders required assessment to be conducted more than 3 months since disease onset or relapse. This study was approved by the SickKids’ Research Ethics Board. All eligible participants were: (i) 5–18.9 years of age and (ii) English speakers or those with at least 2 years of English schooling. Prior to participation in this study, parents provided written informed consent, and children provided assent. When appropriate, participants also provided written informed consent.

Demographic and clinical characteristics are presented in Table 1. Optic neuritis events were characterized as inflammatory episodes with decreased visual acuity lasting more than 24 h, and were associated with: (i) prolonged p100 on visual evoked potential testing (using electroencephalogram), (ii) increased retinal nerve fibre layer thickness on optical coherence tomography measurements (surrogate for optic swelling) or (iii) MRI evidence for inflammation in the corresponding optic nerve. Number of clinical events were defined as neurological events lasting more than 24 h, presumed to be inflammatory, and accompanied by objective neurological findings and/or MRI abnormalities consistent with inflammation and corresponding to these findings. EDSS scores within the last 6 months were utilized to measure the degree of physical disability in children with demyelinating disorders.^64^

**Table 1:** Demographic and clinical characteristics for typically developing children (TDC), children diagnosed with multiple sclerosis (MS) or myelin oligodendrocyte glycoprotein-associated disorders (MOGAD)

	TDC (*n* = 28)	MS (*n* = 18)	MOGAD (*n* = 14)	*P*-value
Sex (*n*)				
Female	17 (60.7%)	12 (67%)	11 (79%)	0.51
Age at assessment (years)				
Mean ± Standard deviation	14.96 ± 2.58 years	16.61 ± 1.09 years	12.14 ± 3.10 years	<0.001
Range	9.00–19.10 years	14.10–18.00 years	7.50–17.30 years	
Age at diagnosis (years)				
Mean ± Standard deviation	-	14.37 ± 1.61 years	9.44 ± 3.44 years	<0.001
Range	-	14.23–17.40 years	2.90–13.80 years	
Time from diagnosis to assessment (years)				
Mean ± Standard deviation	-	2.45 ± 1.64 years	3.04 ± 3.20 years	0.79
Range	-	0.16–6.16 years	0.59–11.71 years	
Number of clinical events				
Mean ± Standard deviation	-	2.06 ± 1.47	2.79 ± 2.33	0.07
Range	-	1–5	1–9	
Number of optic neuritis episodes				
Mean ± Standard deviation	-	0.61 ± 0.85	1.29 ± 0.91	0.12
Range	-	0–2	0–3	
Expanded disability status scale				
Mean ± Standard Deviation	-	1.53 ± 0.87	1.21 ± 0.64	0.26
Range	-	0–4.5	0–1.5	

### MR image acquisition and pre-processing

#### MR image acquisition

MR images were acquired using a 3T Siemens Prisma system (Erlangen, Germany) at SickKids: 3D-T1 MPRAGE Grappa 2 protocol (TE/TR = 3.91/2300 ms, 160 contiguous axial slices, flip angle = 90°, 256 × 224 matrix, FOV = 256 × 224 mm, voxel size = 1 mm ISO and diffusion-weighted (DW) images using a single shot spin-echo sequence (TE/TR = 73/3800 ms, FOV = 244 × 244 mm, 70 slices, slice thickness = 2.0 mm, beta(*b*) = 1000,1600,2600 s/mm^2^). For coregistration between MRI and MEG data, MRI-visible markers were positioned at the same locations as the three MEG fiducial head coils.

#### Children with demyelinating disorders lesion masks

Lesions were segmented at the McConnell Brain Imaging Centre, Montreal Neurological Institute (Montreal, QC), using an automatic change detection naïve Bayesian algorithm. This algorithm classifies tissue based on changes in T1, T2, proton density-weighted and FLAIR images.^65^ After lesions were segmented, lesions were reviewed and manually corrected by trained personnel specializing in demyelinating lesion identification. The resulting lesion masks were used to restrict streamline analyses to normal-appearing white matter for children with demyelinating disorders (as described below).

#### MRI pre-processing

Cortical reconstruction and volumetric segmentation of anatomical T1 images were produced using FastSurfer (v1.1).^66^ T1 anatomical images were motion-corrected before the automated removal of non-brain tissue and segmentation of sub-cortical white matter and deep gray matter structures. Before cortical parcellation was performed using the HCP MMP 1.0 atlas, intensity normalisation, automated topology correction and surface deformation were conducted.^67^ Diffusion-weighted images (DWI) were denoised, eddy current corrected, motion corrected and bias corrected using MRtrix3 (v3.0.2, www.mrtrix.org). DWI from all participants passed quality assurance. All DWI were non-linearly registered to T1 (Talairach) space using advanced normalisation tools (v2.3.5.).^68^

#### DTI and WMTI models

Pre-processed DWI generated DTI metric maps for fractional anisotropy (FA), mean, axial and radial diffusivity (MD, AD and RD). Intracellular and extracellular microstructural parameters were extracted using a WMTI model,^69^ and calculated using weighted linear least squares (https://github.com/orgs/NYU-DiffusionMRI/).^47,70^ The following WMTI metric maps were calculated to assess myelin structure: (i) tortuosity, indicator of myelin volume and (ii) extra-axonal radial diffusivities, sensitive to demyelination and axonal loss and axon structure: (a) axonal water fraction, represents axonal density and (b) intra-axonal axial diffusivity, indicating intra-axonal injury.^47,71^

#### Probabilistic tractography

A multi-shell multi-tissue constrained spherical deconvolution (CSD) approach was utilized to process DWI. DTI maps and whole-brain probabilistic streamlines were generated to extract diffusion metrics from DMN and ECN streamlines from non-lesional white matter (HCP MMP 1.0 atlas^7^). A multi-shell multi-tissue CSD model was used to calculate fibre orientation distributions (FOD).^72-74^ Before streamlines were generated, T1 anatomical images were segmented into tissue types (white matter, cortical grey matter, sub-cortical grey matter, CSF and pathological tissue) to perform anatomically constrained tractography.^75^

Whole-brain probabilistic tractography was conducted for each participant using the iFOD2 algorithm.^76^ Streamlines were generated along paths with high FOD amplitudes and terminated at either sub-cortical grey matter, the grey-white matter interface, or the edge of the image. Propagation of the streamlines stopped once the required number of streamlines was achieved or if the maximum number of seeds was exceeded. Streamline termination was performed by conducting a radial search from the termination point, up to a maximum radius of 4 mm and the endpoint was assigned to the first none-zero node index. Streamlines were constrained to a minimum length of 2.5 mm and a maximum length of 250 mm, with a tensor FA threshold of 0.1. For each participant, 10 million streamlines were generated. Using the spherical-deconvolution informed filtering of tractograms (SIFT) algorithm,^77^ the 10 million streamlines were then filtered to 1 million streamlines to better align the FOD and number of streamlines in each voxel.

Streamlines connecting regions within the DMN (posterior cingulate cortex, medial prefrontal cortex, medial temporal lobe and angular gyrus) and ECN (dorsolateral prefrontal cortex, anterior cingulate cortex and posterior parietal cortex), or between DMN and ECN regions were selected with the following procedure. Inclusion masks were generated for each DMN and ECN region, and exclusion masks were generated for all remaining regions of the HCP MMP 1.0 atlas, the cerebellum and the brainstem to prevent the inclusion of streamlines from these regions. Additionally, for children with demyelinating disorders lesion masks were included as exclusion masks to ensure only normal appearing white matter was analysed. Using these inclusion and exclusion masks, only streamlines connecting regions of the DMN and ECN in normal appearing white matter were extracted. For each participant, DTI and WMTI metric maps were sampled by streamlines connecting regions of the DMN and ECN to calculate mean metric values for each pairwise connection.

### MEG data acquisition and pre-processing

#### MEG recording

Neuromagnetic activity was recorded using a whole-head 151-channel MEG system (CTF Systems Inc., Canada) in a magnetically shielded room while simultaneously recording eye movements (MEG compatible 500 Hz EyeLink1000; SR Research Ltd, Canada) during an interleaved pro- and anti-saccade task (described below). Third-order synthetic gradiometers were used to correct for noise. Continuous MEG data were collected as participants lay supine with stimuli displayed on a screen via an LCD projector with a 60 Hz refresh rate. Eye position was calibrated using a nine-point array covering the visual field. MEG data were recorded at a sampling rate of 600 Hz (samples/second). Prior to MEG data acquisition, each participant was fitted with three fiducial head coils placed on the nasion, left and right pre-auricular areas to localize their head position relative to the MEG sensors. Head position was continuously monitored and recorded before and after each session by sending a small alternating current through these fiducial coils, allowing for the removal of data with more than 10 mm of head motion. Eye movements were tracked with electro-oculograms placed on the left and right temples, and the mastoid process served as a ground reference. MRI-visible fiducial markers were placed at the same nasion, left and right pre-auricular positions to ensure accurate co-registration of MEG and MRI data. The timing of visual stimuli, eye positions and trial markers were synchronized with MEG data collection.

#### MEG pro- and anti-saccade task

Participants completed three blocks of interleaved pro- and anti-saccade trials (120 trials/block, 7 min/block) (Fig. 2A). Each trial began with a circular fixation-instructional cue displayed at the centre of the screen for 1000 ms. A green fixation cue indicated a pro-saccade trial (look towards stimulus location), while a red fixation cue indicated an anti-saccade trial (look away from stimulus). Following a 200 ms gap after the disappearance of the fixation-instructional cue in which no stimuli occurred. Following the gap period, a target stimulus (white circle) appeared 10° horizontally to the left or right side of the central cue. Participants had 1400 ms before the start of the next trial to execute a saccade and re-establish central fixation on the fixation instructional cue. Task and stimulus location were pseudo-randomly interleaved with equal frequency.

#### MEG data pre-processing

All three blocks of the pro- and anti-saccade tasks were first combined using the BrainWave MATLAB toolbox (MathWorks Inc., USA).^78^ MEG data were pre-processed using a custom-built MEG pipeline (https://github.com/MabbottLab/MEGneto) in MATLAB using the Fieldtrip Toolbox.^79^ MEG data were separated into pro- and anti-saccade trials. Continuous raw MEG data were band-pass filtered at 1–150 Hz. A notch filter at 60 Hz and a third-order spatial gradient environmental noise-cancellation was applied. MEG trials were segmented into epochs 2 s before and after stimulus presentation. Trials were removed prior to analysis if they contained excessive head motion (>10 mm), head motion velocity (>15 mm/s), electromagnetic artefacts and muscle movements. Participants were removed from further analyses if they had >15 channels or 20% of trials removed. For the remaining trials, independent component analysis identified and removed heartbeats or eye-blinks contaminating the MEG signal.^80^ Participants were removed from further analyses if they had >5% of channels removed. On average, during the pro-saccade task, participants had 5.67 of channels and 2.57% of components removed during pre-processing; for the anti-saccade task, participants had 5.90 of channels and 2.71% of components removed during pre-processing. Following quality assurance to remove noise in the MEG signal during the pro- and anti-saccade tasks, a sub-sample of 26 typically developing children and 30 children with demyelinating disorders were included in the final task analyses.

Individual time-series sources were reconstructed for the 360 regions of the HCP MMP 1.0 atlas^67^ using a single-shell linearly constrained minimum variance beamforming approach.^81^ To ensure that source locations corresponded across participants, the source model of each participant was normalized to a template source model in MNI space.^82^ MEG data were filtered into canonical frequency bands: theta (4–7 Hz), alpha (8–12 Hz), beta (13–29 Hz), low gamma (30–59 Hz) and high gamma (60–100 Hz). Weighted phase-lag index (wPLI) values were calculated within and between regions of the DMN and ECN to index functional connectivity. This generated a 7 × 7 adjacency matrix for each participant and for each frequency band. wPLI represents the level of synchrony between the neural oscillations of two regions.^83^

### Behavioural analyses

Eye-tracking data were analysed using custom MATLAB software.^84^ A saccade was defined as an eye movement reaching a threshold velocity of 30°/s. Saccades occurring within 90 ms of stimulus appearance were classified as anticipatory and excluded from our analyses. Correct pro-saccades were those directed towards the stimulus and landing within 2° of its location, while correct anti-saccades were those directed away from the stimulus and landing within 2° of the opposite location. Saccade reaction times were measured from the appearance of the stimulus to the first saccade away from fixation that exceeded 30°/s. Trials where the initial saccade was made in the incorrect direction relative to the instruction were classified as direction errors and used to calculate direction error rate.

### Statistical analyses

Independent Kruskal–Wallis tests compared groups on age at assessment, age of clinical diagnosis, time since diagnosis and EDSS; chi-squared tests compared groups on sex, number of clinical events and number of optic neuritis events. Two-sample *t*-tests examined group differences on pro- and anti-saccade reaction times and direction errors. Network Based Statistics (NBS) examined group differences in DMN and ECN structural connectivity and neural synchrony.^85^ NBS controls the family wise error rate during mass-univariate testing. Initially, NBS applies a univariate *t*-threshold to each analysed connection within a network to generate a sparse network of supra threshold connections. Each diffusion metric, WMTI metric and frequency band were initially scanned with a threshold of 0.05, and incrementally increased by 0.05 increments to identify the highest threshold that still produced a significant effect. This method highlights the strongest connections in the network with the greatest significance. After the threshold was determined, permutation testing was conducted to assign a *P*-value that controlled for the family wise error rate for each connection.^85^ A total of 5000 permutations were generated, with each permutation involving the random shuffling of participant’s group membership. For each permutation, the largest number of connections in the network, referred to as the component size, was recorded. For each measure that was found to have a significant NBS result, means and standard deviations of connectivity metrics were calculated for each ROI pair and two-sample *t*-tests examined group differences. All analyses were corrected for multiple comparisons with Bonferroni correction.

As a secondary analysis, to examine the influence of age on task performance and network connectivity of the DMN and ECN, participants were binned into younger (7–14 years) or older age groups (15 + years). These age groups were based on the overall mean age of participants across the groups (MS = 16.61 years, MOGAD = 12.14 years, typically developing children = 14.96 years), which is 14.57 years. Due to the uneven distribution of ages within the patient groups (the MS group included 1 younger and 18 older participants, and the MOGAD group had 12 younger and 1 older participant), MS and MOGAD were combined for a comparative analysis with typically developing children. After combining the patient groups, the typically developing group included 16 younger and 12 older participants, and the children with demyelinating disorders included 13 younger and 18 older participants. The following contrasts assessed the influence of age on task performance and network connectivity: younger and older children with demyelinating disorders, younger and older typically developing children, younger children with demyelinating disorders and younger typically developing children, older children with demyelinating disorders and older typically developing children.

To examine the interaction among oculomotor behaviour, neural activity and structural connectivity, three partial least squares (PLS) path models were built in R (v3.3.2; PLSpm, 5000 bootstraps^86^) for: (i) typically developing children and children with demyelinating disorders, (ii) children diagnosed with MS and (iii) children diagnosed with MOGAD. The accuracy of each model was assessed by examining the relationship between the latent constructs and their associated measures. The latent constructs included: (i) participant, indicated by group status, sex and age at assessment. For our patient models, disease disability was indicated by sex, age at assessment, age at clinical diagnosis, EDSS, number of clinical events and number of optic neuritis events; (ii) structural connectivity, indicated by bilateral diffusion and WMTI metrics (FA, axonal water fraction, tortuosity, intra-axonal axial and mean diffusivities loadings on the latent variable was recoded to be similar to the dimensionality of MD, RD, AD and extra-axonal axial and radial diffusivities); (iii) pro- and anti-saccade functional connectivity, indicated by bilateral high gamma wPLI, left hemisphere theta wPLI, and right hemisphere alpha wPLI (high gamma wPLI loadings on the latent variable was recoded to be similar to the dimensionality of theta and alpha synchrony); and (iv) pro- and anti-saccade reaction times and direction errors, which were our outcome variables.

Cronbach’s alpha and Dillon–Goldstein’s rho examined how well each measure corresponded to their latent construct. The discriminant validity of our measurement models were assessed by examining whether the cross-loading for each measure compared to the other latent constructs were larger than the loadings obtained from measures belonging to other latent constructs. All indicators had a loading >0.8 on their respective latent constructs, except for sex, age at assessment and number of clinical events; thus, they were removed from the model (<0.7). All remaining measures corresponded well with their latent constructs (Cronbach’s alpha >0.8, Dillon-Goldstein’s rho >0.7) and satisfied communality (>0.5).^86^ Discriminant validity was also satisfied as manifest variables had higher cross-loadings (>0.1) with their latent variable blocks compared to other latent variable blocks. The quality of our models was assessed by: (i) the significance of the regression fit (*t*-test); (ii) the *R*^2^ determination coefficients of the endogenous variables; (iii) the average variance extracted; and (iv) the Goodness-of-Fit (GoF). A good fit of our measurement models were identified if: (i) *R*^2^ did not contain zero; (ii) bootstrap percentile confidence intervals for path weights (*β*; 95% percentile); and (iii) the GoF was >0.36.^87^ Statistical analyses codes are located in the supplementary materials.

## Results

### Demographic and clinical comparisons

As shown in Table 1, typically developing children and children with demyelinating disorders did not significantly differ in sex assigned at birth (*ꭕ*^2^_(1,*n*_  _= 60)_ = 1.34, *P* = 0.51), and our patient groups did not differ in their time since clinical diagnosis of MS or MOGAD (*ꭕ*^2^_(1,*n*_  _= 32)_ = 0.07, *P* = 0.79), EDSS (*ꭕ*^2^_(1,*n*_  _= 32)_ = 7.52, *P* = 0.26), the number of clinical events (*ꭕ*^2^_(1,*n*_  _= 32)_ = 11.38, *P* = 0.07) or the number of optic neuritis events (*ꭕ*^2^_(1,*n*_  _= 32)_ = 5.77, *P* = 0.12). However, children diagnosed with MS were older at study enrolment than MOGAD patients (ꭕ^2^_(1,*n*_  _= 32)_ = 17.63, *P* < 0.001), and age at diagnosis was earlier in children diagnosed with MOGAD relative to MS patients (*ꭕ*^2^_(1,*n*_  _= 32)_ = 16.83, *P* < 0.001).

### Similar group saccade performance

Within each group, participants had significantly longer saccade reaction times on the anti- than the pro-saccade tasks (typically developing children: *t*_(1,28)_ = −8.10, *P* < 0.001; MOGAD: *t*_(1,13)_ = −8.03, *P* < 0.001; MS: *t*_(1,15)_ = −6.57, *P* < 0.001; Fig. 2B). In addition, within each group, participants had a greater percentage of direction errors on the anti- than the pro-saccade tasks (typically developing children: *t*_(1,28)_ = −6.19, *P* < 0.001; MOGAD: *t*_(1,13)_ = −4.08, *P* < 0.001; MS: *t*_(1,15)_ = −4.57, *P* < 0.001; Fig. 2C). However, there were no significant group differences on saccade reaction times or direction errors on the pro- or the anti-saccade task between typically developing children and children with demyelinating disorders, or between children diagnosed with MOGAD or MS (all *P*’s > 0.05).

For children with demyelinating disorders, pro-saccade reaction times significantly correlated with age at clinical diagnosis, with longer pro-saccade reaction times found at younger ages of diagnoses (*r* = −0.50, *P* = 0.018; Supplementary Fig. 1). In addition, pro-saccade direction errors significantly correlated with the number of optic neuritis events, with a greater percentage of pro-saccade direction errors found with increased number of optic neuritis events (*r* = 0.44, *P* = 0.043; Supplementary Fig. 1). In comparison, anti-saccade reaction times significantly correlated with EDSS, with longer anti-saccade reaction times found with increased disease disability (*r* = 0.81, *P* = 0.002; Supplementary Fig. 1). Furthermore, anti-saccade direction errors significantly correlated with both age at assessment (*r* = −0.62, *P* = 0.002) and age at clinical diagnosis (*r* = −0.52, *P* = 0.014), with greater anti-saccade direction errors found at both younger ages of assessment and clinical diagnosis (Supplementary Fig. 1).

Pro- and anti-saccade task saccade reaction times and direction errors were assessed for age group differences. There were no significant group differences in saccade reaction times or direction errors on the pro-saccade task between younger and older children with demyelinating disorders, younger and older typically developing children, younger children with demyelinating disorders and younger typically developing children, or between older children with demyelinating disorders and older typically developing children (two-sample *t*-tests; all *P*’s > 0.05). However, younger typically developing children had significantly longer saccade reaction times (*t* = 2.31, *P* = 0.03) and greater direction errors (*t* = 2.41, *P* = 0.02) on the anti-saccade task compared to older typically developing children. No significant group differences were found for saccade reaction times or direction errors on the anti-saccade task between younger and older children with demyelinating disorders, younger children with demyelinating disorders and younger typically developing children, or between older children with demyelinating disorders and older typically developing children (two-sample *t*-tests; all *P*’s > 0.05).

### Structural connectivity compromised in children with demyelinating disorders

**Figure 3:**
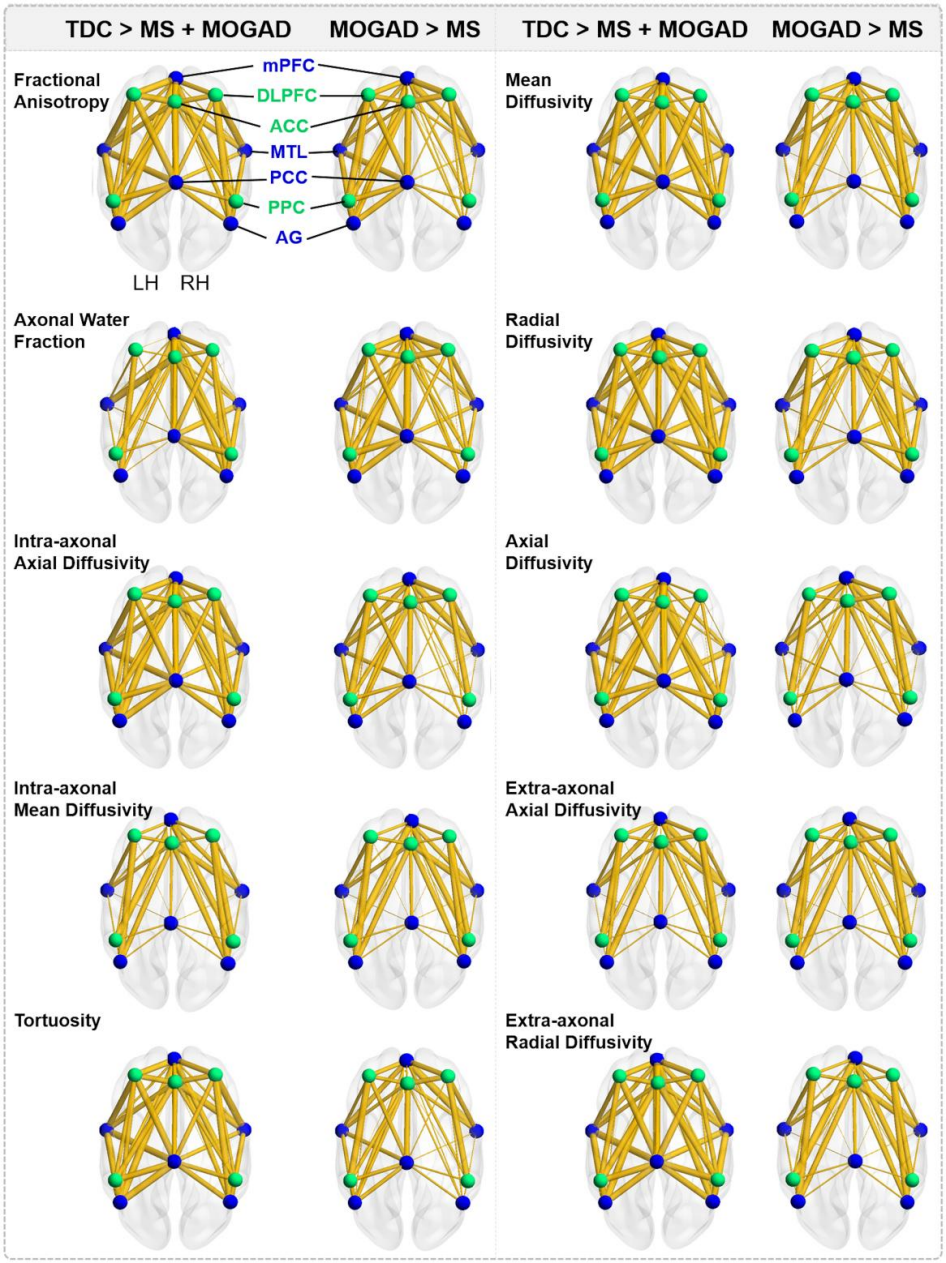
**Default mode and ECN network connections with compromised structural connectivity for children with demyelinating disorders compared to typically developing children.** Values generated using *t*-test in network-based statistics, for 26 typically developing children, 18 children with MS and 14 children with MOGAD, with value <3 denoted as non-significant. Wider connections depict more significant differences between groups. This figure demonstrates that children with demyelinating disorders had significant DMN and ECN myelin and axon compromise relative to typically developing children. Furthermore, children diagnosed with MS displayed greater structural compromise than children diagnosed with MOGAD. *Note.* PCC, posterior cingulate cortex; mPFC, medial prefrontal cortex; MTL, medial temporal lobe; AG, angular gyrus; DLPFC, dorsolateral prefrontal cortex; ACC, anterior cingulate cortex; PPC, posterior parietal cortex; TDC, typically developing children; MOGAD, children diagnosed with myelin oligodendrocyte glycoprotein-associated disorders; MS, children diagnosed with multiple sclerosis; LH, left hemisphere; RH, right hemisphere.

Children with demyelinating disorders showed significant bilateral DMN and ECN myelin and axon compromise compared to typically developing children (Fig. 3). Specifically, children with demyelinating disorders displayed significantly lower FA and higher MD, RD and AD bilaterally than typically developing children (NBS *t*-test; all *P’s* < 0.05). Similar results were found when examining DMN and ECN intracellular and extracellular microstructural parameters that were extracted using a WMTI model to provide more specific inferences on myelin and axon structure. Compared to children with demyelinating disorders, typically developing children displayed significantly higher axonal water fraction, intra-axonal axial and mean diffusivities and tortuosity (NBS *t*-test; all *P’s* < 0.01). In contrast, typically developing children had significantly lower extra-axonal axial and radial diffusivities relative to children with demyelinating disorders (NBS *t*-test; all *P’s* < 0.01).

Among participants with demyelinating disorders, children diagnosed with MOGAD displayed higher FA, axonal water fraction, intra-axonal axial and mean diffusivities, and tortuosity and lower MD, RD, AD, extra-axonal axial and radial diffusivities relative to children with MS (NBS *t*-test; all *P’s* < 0.01). Across both MS and MOGAD diagnoses, white matter organisation metrics (FA, MD, RD and AD) and myelin and axon related indices (axonal water fraction, extra-axonal radial diffusivity, intra-axonal axial and mean diffusivities and tortuosity) significantly correlated with age at clinical diagnosis, with greater myelin and axon compromise found at younger ages of diagnoses.

DMN and ECN myelin and axon microstructure was assessed for age group differences with the following contrasts: younger and older children with demyelinating disorders, younger and older typically developing children, younger children with demyelinating disorders and younger typically developing children, older children with demyelinating disorders and older typically developing children (younger: 7–14 years, older: 15 + years). NBS revealed that older children with demyelinating disorders displayed significantly lower FA and higher MD, RD and AD bilaterally compared to older typically developing children (NBS *t*-test; all *P’s* < 0.01). Similarly, younger children with demyelinating disorders showed significantly lower FA and higher MD, RD and AD bilaterally compared to younger typically developing children (NBS *t*-test; all *P’s* < 0.01). No significant group differences were found when comparing younger and older typically developing children, or younger and older children with demyelinating disorders (NBS *t*-test; all *P’s* > 0.05). When examining DMN and ECN intracellular and extracellular microstructural parameters, older typically developing children displayed significantly higher bilaterally axonal water fraction, intra-axonal axial and mean diffusivities and tortuosity compared to older children with demyelinating disorders (NBS *t*-test; all *P’s* < 0.01). In addition, older typically developing children had significantly lower bilaterally extra-axonal axial and radial diffusivities relative to older children with demyelinating disorders (NBS *t*-test; all *P’s* < 0.01). No significant group differences were found in DMN and ECN intracellular and extracellular microstructural parameters between younger children with demyelinating disorders and younger typically developing children, between younger and older children with demyelinating disorders, or between younger and older typically developing children (NBS *t*-test; all *P’s* > 0.05).

### Neural synchrony perturbed in children with demyelinating disorders

**Figure 4:**
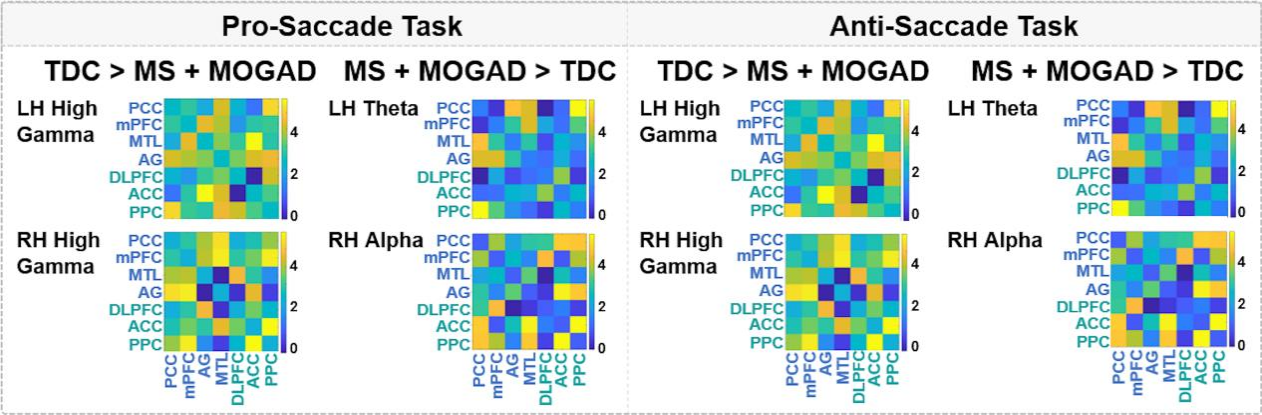
**Default mode and ECN network connections with compromised pro- and anti-saccade functional connectivity for children with demyelinating disorders compared to typically developing children.** Values generated using *t*-test in network-based statistics, for 26 typically developing children, 17 children with MS and 13 children with MOGAD, with value <3 denoted as non-significant. The connectivity matrices presented in this figure depict group differences in mean node connectivity strength of pairwise associations for each measure, with brighter connectivity colours indicating a larger group difference in mean metric values. There were no significant differences between patient groups in network neural synchrony. PCC, posterior cingulate cortex; mPFC, medial prefrontal cortex; MTL, medial temporal lobe; AG, angular gyrus; DLPFC, dorsolateral prefrontal cortex; ACC, anterior cingulate cortex; PPC, posterior parietal cortex; TDC, typically developing children; MOGAD, children diagnosed with myelin oligodendrocyte glycoprotein-associated disorders; MS, children diagnosed with multiple sclerosis; LH, left hemisphere; RH, right hemisphere.

During the pro- and anti-saccade tasks, children with demyelinating disorders displayed lower bilateral high gamma, higher left hemisphere theta and higher right hemisphere alpha synchrony in the DMN and ECN relative to typically developing children (NBS *t*-test; *P* < 0.05) (Fig. 4). There were no significant differences in DMN and ECN neural synchrony during the pro- and anti-saccade tasks between children diagnosed with MOGAD or MS (NBS *t*-test; all *P’s* > 0.05). Furthermore, DMN and ECN neural synchrony was not correlated with age at clinical diagnosis (all *P’s* > 0.05); however, our trends suggest that younger ages of diagnoses may be associated with lower DMN and ECN neural synchrony.

When examining the influence of age, NBS revealed that older children with demyelinating disorders exhibited lower bilateral DMN and ECN high gamma, higher left hemisphere theta and higher right hemisphere alpha synchrony compared to older typically developing children (NBS *t*-test; *P* < 0.05). In addition, older children with demyelinating disorders had significantly greater right hemisphere alpha synchrony during the pro- and anti-saccade tasks relative to younger children with demyelinating disorders (NBS *t*-test; *P* < 0.05). No significant group differences were found in bilateral high gamma, left hemisphere theta or right hemisphere alpha neural synchrony during the pro- and anti-saccade tasks between younger children with demyelinating disorders and younger typically developing children, or between younger and older typically developing children (NBS *t*-test; all *P’s* > 0.05).

### Adaptive functional reorganisation in response to white matter changes

**Figure 5:**
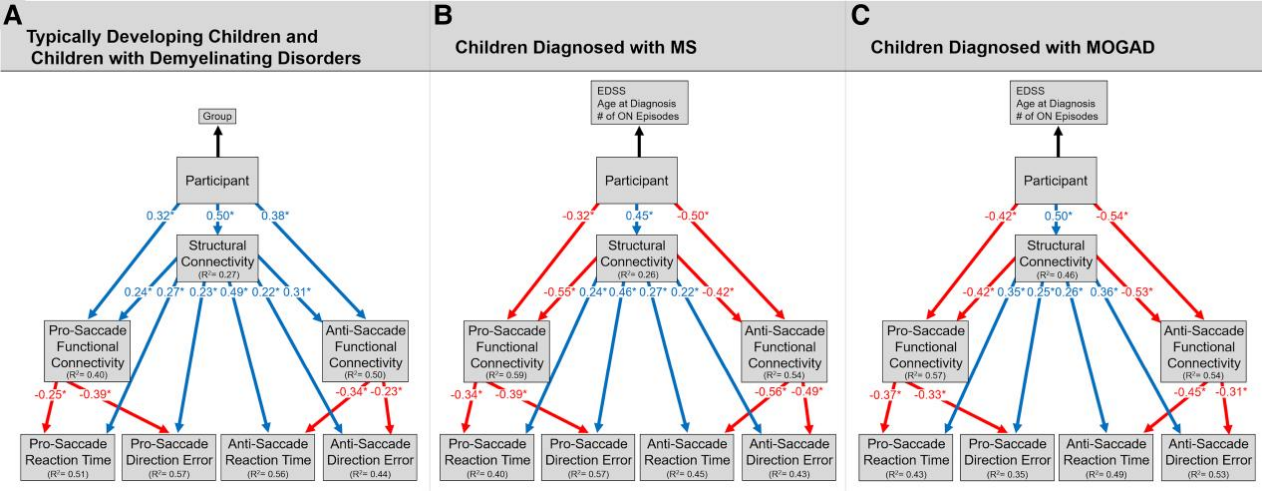
**Adaptive functional reorganisation in response to white matter changes resulting in cognitive preservation for children with demyelinating disorders.** Results from our PLS path modelling for 26 typically developing children, 17 children with MS and 13 children with MOGAD (**A**) indicates that children with demyelinating disorders had greater myelin and axon compromise of the DMN and ECN networks relative to typically developing children. In turn, increased structural compromise directly predicted longer pro- and anti-saccade reaction times and greater pro- and anti-saccade direction errors. However, in our model both children with demyelinating disorders and increased myelin and axon compromise of the DMN and ECN separately predicted greater pro- and anti-saccade DMN and ECN functional connectivity, which highlights potential adaptive functional reorganisation of these networks in response to structural compromise. In turn, increased DMN and ECN functional connectivity predicted faster saccade reaction times and fewer pro- and anti-saccade direction errors. To examine the influence of disease disability on DMN and ECN structure-function connectivity and saccade performance, separate PLS path models were conducted for children with demyelinating disorders. For both children diagnosed with MS (**B**) and MOGAD (**C**), we found that increased disease disability, highlighted by EDSS, age at diagnosis and number of optic neuritis episodes, predicted greater myelin and axon compromise of the DMN and ECN. In turn, increased structural compromise directly predicted longer pro- and anti-saccade reaction times and greater pro- and anti-saccade direction errors. Increased disease disability and increased myelin and axon compromise of the DMN and ECN both directly predicted decreased pro- and anti-saccade DMN and ECN neural synchrony. In turn, decreased DMN and ECN functional connectivity predicted longer pro- and anti-saccade reaction times and greater pro- and anti-saccade direction errors. MOGAD, children diagnosed with myelin oligodendrocyte glycoprotein-associated disorders; MS, children diagnosed with multiple sclerosis; ON, optic neuritis; EDSS, expanded disability status scale; * = *P* < 0.05.

Our PLS path model revealed that children with demyelinating disorders had significantly greater myelin and axon compromise of the DMN and ECN relative to typically developing children (Fig. 5A, Tables 2 and 3). This structural compromise predicted greater DMN and ECN functional connectivity, longer pro- and anti- saccade reaction times and greater direction errors (*P* < 0.05). In addition, participant directly predicted pro- and anti-saccade functional connectivity, which predicted shorter saccade reaction times and fewer direction errors (*P* < 0.05). The indirect effect of structural connectivity on saccade reaction time through functional connectivity was significant for both the pro-saccade (*β* = 0.42) and anti-saccade task (*β* = 0.47). Similarly, the indirect effect of structural connectivity on direction errors through functional connectivity was also significant for both the pro-saccade (*β* = 0.39) and anti-saccade task (*β* = 0.49). The GoF for our model was 0.44 (>0.36).

**Table 2:** PLSs path modelling results to examine how neural function may compensate for structural compromise in children with demyelinating disorders and preserve cognition

Path	*β*	CI_95_	*t*-value	*P*-value
Typically developing children and children with demyelinating disorders				
Participant → Structural connectivity	0.50	0.38, 0.64	4.05	0.00018
Participant → Pro-saccade functional connectivity	0.32	−0.31, 0.51	2.87	<0.001
Participant → Anti-saccade functional connectivity	0.38	−0.21, 0.55	1.39	<0.001
Structural connectivity → Pro-saccade functional connectivity	0.24	−0.37, 0.47	3.60	0.001
Structural connectivity → Pro-saccade reaction time	0.27	0.03, 0.49	1.95	0.02
Structural connectivity → Pro-saccade direction errors	0.23	0.13, 0.61	1.29	0.03
Structural connectivity → Anti-saccade functional connectivity	0.31	0.03, 0.66	1.95	<0.001
Structural connectivity → Anti-saccade reaction time	0.49	0.37, 0.66	3.92	0.0003
Structural connectivity → Anti-saccade direction errors	0.22	0.07, 0.45	1.34	0.03
Pro-saccade functional connectivity → Pro-saccade reaction time	−0.25	−0.17, 0.32	−1.79	0.002
Pro-saccade functional connectivity → Pro-saccade direction errors	−0.39	−0.74, 0.32	−2.26	0.03
Anti-saccade functional connectivity → Anti-saccade reaction time	−0.34	−0.23, 0.30	−1.15	<0.001
Anti-saccade functional connectivity → Anti-saccade direction errors	−0.23	−0.47, 0.42	−1.34	0.03
Children diagnosed with multiple sclerosis				
Participant → Structural connectivity	0.45	0.36, 0.74	2.39	0.03
Participant → Pro-saccade functional connectivity	−0.32	−0.31, 0.51	2.07	<0.001
Participant → Anti-saccade functional connectivity	−0.50	−0.21, 0.55	2.49	<0.001
Structural connectivity → Pro-saccade functional connectivity	−0.55	−0.37, 0.47	3.51	0.002
Structural connectivity → Pro-saccade reaction time	0.24	0.03, 0.49	1.95	0.02
Structural connectivity → Pro-saccade direction errors	0.46	0.03, 0.73	1.94	0.02
Structural connectivity → Anti-saccade functional connectivity	−0.42	0.37, 0.66	4.17	0.0003
Structural connectivity → Anti-saccade reaction time	0.27	0.03, 0.66	1.95	<0.001
Structural connectivity → Anti-saccade direction errors	0.22	0.37, 0.66	1.18	0.03
Pro-saccade functional connectivity → Pro-saccade reaction time	−0.34	−0.17, 0.32	−3.13	0.004
Pro-saccade functional connectivity → Pro-saccade direction errors	−0.39	−0.79, 0.53	−3.56	0.002
Anti-saccade functional connectivity → Anti-saccade reaction time	−0.56	−0.71, 0.30	−2.07	<0.001
Anti-saccade functional connectivity → Anti-saccade direction errors	−0.49	−0.87, 0.72	−2.53	0.02
Children diagnosed with myelin oligodendrocyte glycoprotein-associated disorders				
Participant → Structural connectivity	0.50	−0.44, 0.93	2.91	0.02
Participant → Pro-saccade functional connectivity	−0.42	−0.63, 0.76	1.37	0.04
Participant → Anti-saccade functional connectivity	−0.54	−0.47, 0.24	2.93	0.02
Structural connectivity → Pro-saccade functional connectivity	−0.42	−0.86, 0.32	2.71	0.02
Structural connectivity → Pro-saccade reaction time	0.35	−0.28, 0.58	2.73	0.04
Structural connectivity → Pro-saccade direction errors	0.25	−0.36, 0.67	2.75	0.03
Structural connectivity → Anti-saccade functional connectivity	−0.53	−0.72, 0.65	2.99	0.02
Structural connectivity → Anti-saccade reaction time	0.26	−0.50, 0.95	1.23	0.25
Structural connectivity → Anti-saccade direction errors	0.36	−0.81, 0.98	5.21	0.61
Pro-saccade functional connectivity → Pro-saccade reaction time	−0.37	−0.80, 0.56	−2.53	0.03
Pro-saccade functional connectivity → Pro-saccade direction errors	−0.33	−0.89, 0.42	−2.44	0.04
Anti-saccade functional connectivity → Anti-saccade reaction time	−0.45	−0.71, 0.18	−2.84	0.02
Anti-saccade functional connectivity → Anti-saccade direction errors	−0.31	−0.92, 0.37	−2.99	0.02

**Table 3:** Coefficients of determination (R2) for each latent variable in our PLSs path model

Latent variable	*R* ^2^	CI_95_	Average variance extracted
Typically developing children and children with demyelinating disorders			
Structural connectivity	0.47	0.23, 0.67	0.68
Pro-saccade functional connectivity	0.40	0.24, 0.68	0.62
Anti-saccade functional connectivity	0.50	0.24, 0.63	0.64
Pro-saccade reaction time	0.51	0.23, 0.80	-
Anti-saccade reaction time	0.56	0.43, 0.84	-
Pro-saccade direction errors	0.57	0.16, 0.93	-
Anti-saccade direction errors	0.44	0.18, 0.64	-
Children diagnosed with multiple sclerosis			
Structural connectivity	0.26	0.14, 0.59	0.76
Pro-saccade functional connectivity	0.59	0.20, 0.79	0.72
Anti-saccade functional connectivity	0.54	0.13, 0.77	0.71
Pro-saccade reaction time	0.40	0.15, 0.78	-
Anti-saccade reaction time	0.45	0.17, 0.72	-
Pro-saccade direction errors	0.57	0.23, 0.72	-
Anti-saccade direction errors	0.43	0.14, 0.68	-
Children diagnosed with myelin oligodendrocyte glycoprotein-associated disorders			
Structural connectivity	0.46	0.24, 0.92	0.74
Pro-saccade functional connectivity	0.57	0.37, 0.95	0.69
Anti-saccade functional connectivity	0.54	0.46, 0.94	0.70
Pro-saccade reaction time	0.43	0.29, 0.82	-
Anti-saccade reaction time	0.35	0.31, 0.89	-
Pro-saccade direction errors	0.49	0.22, 0.69	-
Anti-saccade direction errors	0.53	0.27, 0.82	-

When separately examining children diagnosed with MS, our model revealed that greater disease disability, proxied by EDSS, age at diagnosis and number of optic neuritis episodes, predicted greater myelin and axon compromise of the DMN and ECN (Fig. 5B, Tables 2 and 3). This structural compromise predicted decreased DMN and ECN functional connectivity, longer saccade reaction times and greater direction errors for both the pro- and anti-saccade tasks (*P* < 0.05). Increased disease disability also predicted decreased functional connectivity, which in turn, predicted longer saccade reaction times and greater direction errors (*P* < 0.05). The indirect effect of structural connectivity on saccade reaction time through functional connectivity was significant for the pro-saccade (*β* = 0.42) and anti-saccade task (*β* = 0.47). Similarly, the indirect effect of structural connectivity on direction errors through functional connectivity was also significant for both the pro-saccade (*β* = 0.46) and anti-saccade task (*β* = 0.39). The GoF for our model was 0.52. Similar PLS path model results were found for children diagnosed with MOGAD (Fig. 5C, Tables 2 and 3); the GoF for our model was 0.54.

## Discussion

This study sought to evaluate the interplay among structural organisation, neural activity and oculomotor behaviour in children with MS and MOGAD. We demonstrated that, despite the similar behavioural task performance, children with demyelinating disorders showed: DMN and ECN myelin and axon compromise, and lower bilateral high gamma, higher left hemisphere theta, and higher right hemisphere alpha synchrony during the pro and anti-saccade tasks compared to typically developing children. Among participants with demyelinating disorders, children diagnosed with MS had greater structural compromise relative to children with MOGAD; however, there were no significant group differences in neural synchrony. For both patient groups, increased disease disability, highlighted by EDSS, age at diagnosis and number of optic neuritis episodes, predicted greater myelin and axon compromise, which in turn predicted longer pro- and anti-saccade reaction times and greater direction errors. However, increased structural compromise also predicted increased pro- and anti-saccade DMN and ECN functional connectivity, highlighting potential adaptive functional reorganisation in response to structural compromise. In turn, increased functional connectivity predicted faster saccade reaction times and fewer direction errors. These findings indicate that despite the DMN and ECN structural compromise in children with demyelinating disorders, potential compensatory mechanisms, indicated by increased alpha and theta synchrony, may be contributing to the similar behavioural group performance.

Consistent with previous diffusion MRI studies, children with demyelinating disorders displayed compromise of non-lesioned normal-appearing white matter relative to typically developing children.^10,69,88^ Children diagnosed with MOGAD or MS displayed lower white matter organisation, tortuosity, axonal water fraction and intra-axonal diffusivity, as well as increased extra-axonal radial diffusivity compared to typically developing children. Furthermore, consistent with previous findings showcasing pathological differences between MS and MOGAD, children diagnosed with MS displayed significantly lower structural connectivity relative to children diagnosed with MOGAD.^89,90^ Axonal water fraction and intra-axonal axial diffusivity are both thought to be potential markers of axonal loss and intra-axonal injury, respectively.^46,48,91^ Thus, decreased axonal water fraction and intra-axonal axial diffusivity in children with demyelinating disorders relative to typically developing children reflect axonal density changes due to injury. Furthermore, tortuosity and extra-axonal radial diffusivities are hypothesized to respectively reflect myelin volume and myelin integrity.^46,48,69^ Decreased tortuosity and increased extra-axonal radial diffusivities may reflect greater demyelination within the DMN and ECN for children with demyelinating disorders relative to typically developing children. Taken together, these findings indicate the impact of demyelination on myelin and axon structure and highlight the role of axonal injury that accompanies myelin injury in children with demyelinating disorders. Furthermore, as shown in Fig. 5B and C, increased motor disability, highlighted by EDSS, age at diagnosis and number of optic neuritis episodes, predicted increased myelin and axon compromise. In turn, increased structural compromise of the DMN and ECN predicted longer pro- and anti-saccade reaction times and greater direction errors. However, increased structural compromise also predicted increased pro- and anti-saccade functional connectivity of the DMN and ECN, which in turn predicted faster pro- and anti-saccade reaction times and fewer direction errors (Fig. 5A). Building upon previously reported results, these findings suggest that increased functional connectivity may be necessary to compensate for structural compromise of the DMN and ECN and preserve cognitive abilities.^38^

Critically, increased functional connectivity of the DMN and ECN was not only influenced by structural compromise, but also by group status, with children with demyelinating disorders predicting greater theta and alpha synchrony and lower high gamma synchrony relative to typically developing children. There were no significant differences in neural synchrony between children diagnosed with MOGAD or MS. These findings indicate that not all changes to functional connectivity are solely due to impacts from structural compromise. Rather, our findings support previous work suggesting that cognition is sub-served by coherently oscillating neuronal populations that produce temporal windows for communication.^2-7^ It is also important to note that functional connectivity is a dynamic and complex phenomenon, which makes it difficult to determine whether these deviations in neural synchrony represent adaptive compensation or pathological changes. However, even though we cannot derive causal conclusions from our findings due to the cross-sectional nature of our study, our results support our hypothesis that cognitive preservation may be due to an adaptive functional reorganisation in response to white matter changes. This cognitive preservation may be due to increased neural synchrony among key regions that support higher-order cognitive processes, which is highlighted by increased alpha and theta synchrony of the DMN and ECN. Specifically, alpha and theta synchrony have been found to be related to complementary aspects of cognitive control during the pro- and anti-saccade tasks.^92,93^ Suppressing irrelevant information has been linked to alpha synchrony,^94,95^ and prioritizing relevant information has been hypothesized to be facilitated by theta synchrony.^96,97^ Importantly, as demonstrated by our findings, both alpha and theta synchrony do not occur in isolation, and are often accompanied by activity in higher frequency bands, in particular gamma frequencies, which have been hypothesized to reflect the maintenance of neuronal representations.^85,86^ The modulation of local high-frequency activity through the phase of a slower rhythm may be a putative mechanism for the synchronisation of fast oscillations across distant regions of our two networks that is necessary to maintain typical cognition.^98^ We propose that for children with demyelinating disorders, decreases in high gamma synchrony may reflect difficulties in the neuronal maintenance of visual representations, but this is compensated for by an increased reliance on attentional control and central executive processing, as reflected by increases in alpha and theta synchrony.

In considering our analyses and results, it is worth noting some considerations related to white matter microstructure and brain development that requires further investigation. Paediatric demyelinating events occur during key periods of myelination and network neural maturation^13,14,16^; therefore, longitudinal studies are key to exploring the interactions among progressive brain pathology, neural plasticity and potential compensatory mechanisms and how these mechanisms may change during the progression of demyelinating disorders or relapse/remit phases. These longitudinal studies are important to determining whether functional changes in response to structural compromise are ‘adaptive’ (i.e. protecting against or limiting cognitive impairments) or ‘maladaptive’ (i.e. contributing to cognitive impairments). For example, increased neural synchrony between networks may compensate for structural compromise leading to typical cognitive performance; however, it is also plausible that increased network neural synchrony may be a downstream maladaptive consequence of structural compromise rather than a compensation for this compromise. It is also important to investigate if there are differential effects on distinct cognitive domains, beyond what we measured in this study using the pro- and anti-saccade tasks. Addressing these and related questions will further the understanding of the underlying mechanisms of cognitive preservation in children with demyelinating disorders and can be used to identify children who may be at high risk for developing cognitive impairments.

The results of this work are primarily limited by the characteristics of the clinical population studied. Although our sample was large for an imaging study with a clinical population, the group sample sizes limited our ability to examine individual differences that may impact compensatory links in structure-function coupling on cognition. Furthermore, there was heterogeneity in our patient demographics. For example, our patient sample had a wide age range, and even though there were no significant group differences on age between typically developing children and children with demyelinating disorders, there are maturational changes that occurs in the brain during childhood and adolescence.^99^ Additional research is necessary to explore the influence of brain development on neural network connectivity and cognitive performance using longitudinal and within-subject experimental designs. Furthermore, our patient groups had heterogeneous clinical characteristics and disease manifestations which may be related to varying patterns of network connectivity. Controlling these medical variables is important to further examine how white matter plasticity is associated with cognitive preservation in children with demyelinating disorders.

Despite these limitations, results from this study provide key insights into potential factors contributing to the preservation of cognitive abilities in children with demyelinating disorders. We show that during the presence of task demands, adaptive functional reorganisation of the DMN and ECN takes place to compensate for structural compromise of these networks and preserve cognitive performance. The relationship between pro- and anti-saccade behaviour and clinical characteristics suggest that this eye tracking metrics may have utility in tracking disease progression and functional recovery in children with demyelinating disorders. Longitudinal studies integrating multiple neuroimaging modalities are key to understanding how eye tracking behaviours change during the progression of demyelinating disorders. These studies can also help assess the usefulness of these behaviours as indicators of disease progression, treatment effectiveness and the brain’s compensatory responses. Further understanding these compensatory neural mechanisms in children with demyelinating disorders could pave the way for the development of targeted therapeutic interventions aimed at enhancing these mechanisms, ultimately improving cognitive outcomes for affected individuals.

## Supplementary Material

fcae353_Supplementary_Data

## Data Availability

Data supporting findings of this study are available on request from the corresponding author. Data are not publicly available due to privacy or ethical restrictions.
