# Supplementary material for: Compensatory mechanisms amidst demyelinating disorders: insights into cognitive preservation
Source: Brain Commun. 2024 Nov 12;6(6):fcae353. doi: 10.1093/braincomms/fcae353 (PMC11554762; doi:10.1093/braincomms/fcae353)
Supplement: fcae353_Supplementary_Data [file fcae353_supplementary_data.pdf]

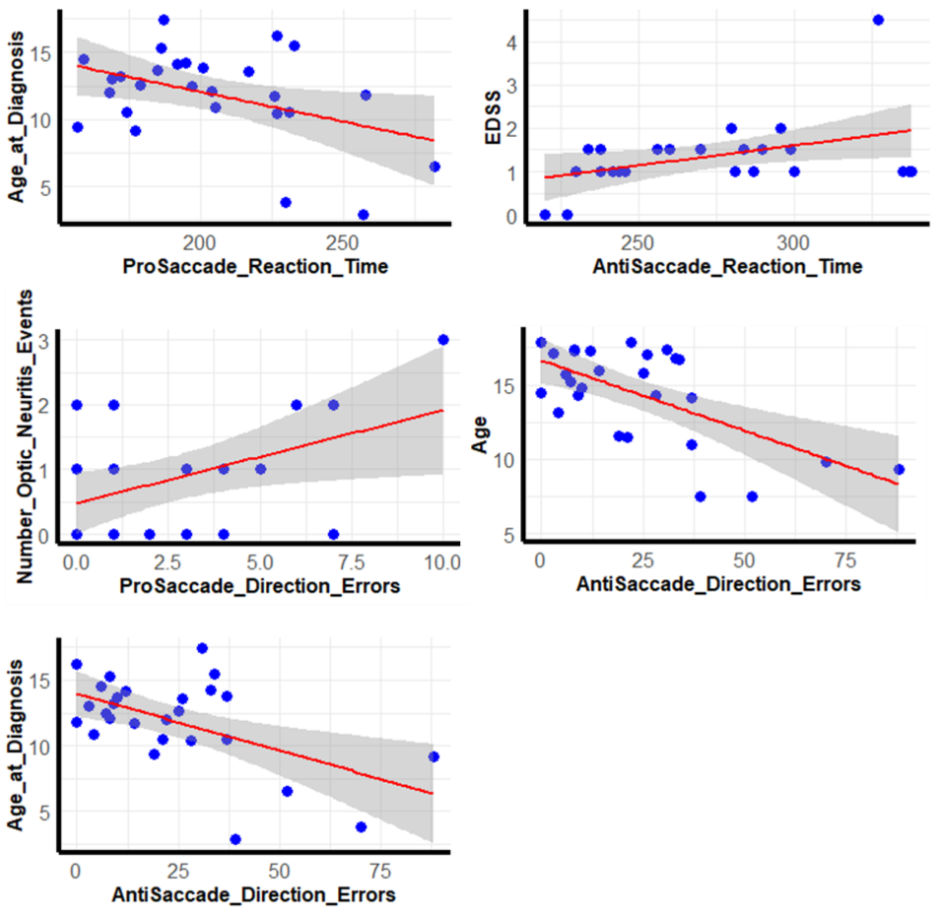

Supplementary Figure 1. Correlations between pro- and anti-saccade performance with clinical parameters for children with demyelinating disorders. Each data point in these figures represents a participant with a demyelinating disorder (*n* = 32). For children with demyelinating disorders, pro-saccade reaction times significantly correlated with age at clinical diagnosis, with longer pro-saccade reaction times found at younger ages of diagnoses (r = -0.50, P = 0.018). In addition, pro-saccade direction errors significantly correlated with the number of optic neuritis events, with a greater percentage of pro-saccade direction errors found with increased number of optic neuritis events (r = .44, P = 0.043). In comparison, anti-saccade reaction times significantly correlated with Expanded Disability Status Scale (EDSS), with longer anti-saccade reaction times found with increased disease disability (r = 0.81, P = 0.002). Furthermore, anti-saccade direction errors significantly correlated with both age at assessment (r = -0.62, P = 0.002) and age at clinical diagnosis (r = -0.52, P = 0.014), with greater anti-saccade direction errors found at both younger ages of assessment and clinical diagnosis.

Statistical Analyses Code:

```
# Load necessary libraries
library(readxl)  # For reading Excel files
library(dplyr)   # For data manipulation
library(ggplot2) # For plotting
library(reshape2) # For reshaping data
library(plspm)  # For PLS Path Modeling

# Load the Excel file
demographics <- read_excel("AlDahhanetal_BrainComm.xlsx")

# Select only the numeric columns to correlate
numeric_data <- demographics %>%
  select(Age, PS_cnt_reg_err, PS_mean_RegCor_SRT, AS_cnt_reg_err,
AS_mean_RegCor_SRT,
         Age_at_Diagnosis, Time_from_Diagnosis_to_Assessment, EDSS,
         Number_Clinical_Events, Number_Optic_Neuritis_Events)

# Calculate Pearson correlation matrix
cor_matrix <- cor(numeric_data, use = "pairwise.complete.obs")

# Perform pairwise correlation tests (p-values)
cor_test_results <- list()
for(i in 1:ncol(numeric_data)) {
  for(j in i:ncol(numeric_data)) {
    if(i != j) {
      cor_test <- cor.test(numeric_data[[i]], numeric_data[[j]])
      cor_test_results[[paste(names(numeric_data)[i], names(numeric_data)[j], sep = " vs ")]] <-
cor_test
    }
  }
}

# Calculate the total number of tests
n_tests <- length(cor_test_results)

# Apply Bonferroni correction to p-values
bonferroni_corrected_results <- lapply(cor_test_results, function(test) {
  p_adj <- p.adjust(test$p.value, method = "bonferroni", n = n_tests)
  list(correlation = test$estimate, p.value = p_adj)
})

# Extract significant correlations after Bonferroni correction
significant_correlations <- lapply(bonferroni_corrected_results, function(test) {
  if (test$p.value < 0.05) {
    list(correlation = test$correlation, p.value = test$p.value)
```

```
  } else {
    NULL
  }
})

# Filter out NULLs (non-significant results)
significant_correlations <- Filter(Negate(is.null), significant_correlations)

# Create a data frame for plotting significant correlations
significant_df <- data.frame(
  variable1 = sapply(names(significant_correlations), function(x) strsplit(x, " vs ")[[1]][1]),
  variable2 = sapply(names(significant_correlations), function(x) strsplit(x, " vs ")[[1]][2]),
  correlation = sapply(significant_correlations, function(x) x$correlation)
)

# Display the significant correlations
print(significant_df)

# Reshape data for heatmap
heatmap_data <- reshape2::melt(cor_matrix)
colnames(heatmap_data) <- c("Variable1", "Variable2", "Correlation")
heatmap_data$Significant <- ifelse(paste(heatmap_data$Variable1, heatmap_data$Variable2,
sep = " vs ") %in% names(significant_correlations), "Yes", "No")

# Plot heatmap
ggplot(heatmap_data, aes(x = Variable1, y = Variable2, fill = Correlation)) +
  geom_tile(color = "white") +
  scale_fill_gradient2(low = "blue", high = "red", mid = "white", midpoint = 0, limit = c(-1, 1)) +
  theme_minimal() +
  theme(axis.text.x = element_text(angle = 45, hjust = 1)) +
  labs(title = "Correlation Heatmap with Significant Correlations Highlighted",
       fill = "Correlation")

# Function to create scatterplot with trend line, r and p-values
create_scatterplot <- function(data, x, y) {
  # Calculate correlation
  cor_test <- cor.test(data[[x]], data[[y]], method = "pearson")
  r_value <- round(cor_test$estimate, 2)
  p_value <- round(cor_test$p.value, 3)

  # Create scatterplot with trend line
  plot <- ggplot(data, aes_string(x = x, y = y)) +
    geom_point(color = "blue", size = 3) +
    geom_smooth(method = "lm", se = TRUE, color = "red") +
    labs(title = paste("Scatterplot of", x, "vs.", y),
         subtitle = paste("r =", r_value, "p =", p_value),
         x = x,
```

```
      y = y) +
    theme_minimal() +
    theme(
      axis.title = element_text(size = 12, face = "bold"),  # Increase axis title size
      axis.text = element_text(size = 12),                  # Increase axis text size
      axis.line = element_line(size = 1.5),                 # Make axis lines thicker
      plot.title = element_text(size = 12, face = "bold"), # Increase title size
      plot.subtitle = element_text(size = 12)              # Increase subtitle size
    )

  return(plot)
}

# Generate and print scatterplots for the specified comparisons
plot1 <- create_scatterplot(demographics, "ProSaccade_Reaction_Time", "Age_at_Diagnosis")
plot2 <- create_scatterplot(demographics, "ProSaccade_Direction_Errors",
"Number_Optic_Neuritis_Events")
plot3 <- create_scatterplot(demographics, "AntiSaccade_Reaction_Time", "EDSS")
plot4 <- create_scatterplot(demographics, "AntiSaccade_Direction_Errors",
"Age_at_Diagnosis")
plot5 <- create_scatterplot(demographics, "AntiSaccade_Direction_Errors", "Age")

# Print the plots
print(plot1)
print(plot2)
print(plot3)
print(plot4)
print(plot5)

# Age Comparisons

# Convert the 'Group' variable to a factor
demographics$group <- factor(demographics$Group, levels = c("Healthy Controls", "Patients"))
demographics$group <- factor(demographics$Group_Age, levels = c("Younger Controls",
"Younger Patients"))
demographics$group <- factor(demographics$Group_Age, levels = c("Older Controls", "Older
Patients"))
demographics$group <- factor(demographics$Group_Age, levels = c("Younger Controls",
"Older Controls"))
demographics$group <- factor(demographics$Group_Age, levels = c("Younger Patients", "Older
Patients"))

# List of demographic variables to test
demo_vars <- c("PS_cnt_reg_err", "PS_mean_RegCor_SRT", "AS_cnt_reg_err",
"AS_mean_RegCor_SRT")
```

```
# Perform t-tests and store results
t_test_results <- lapply(demo_vars, function(var) {
  formula <- as.formula(paste(var, "~ group"))
  t_test <- t.test(formula, data = demographics, var.equal = TRUE)  # Assuming equal variances;
use var.equal = FALSE if not
  list(variable = var, estimate = t_test$estimate, p.value = t_test$p.value, statistic =
t_test$statistic, conf.int = t_test$conf.int)
})

# Extract p-values and apply Bonferroni correction
t_test_pvalues <- sapply(t_test_results, function(x) x$p.value)
t_test_bonferroni <- p.adjust(t_test_pvalues, method = "bonferroni", n = length(t_test_pvalues))

# Combine results into a data frame
results_df <- do.call(rbind, lapply(seq_along(demo_vars), function(i) {
  result <- t_test_results[[i]]
  data.frame(variable = result$variable, mean_diff = diff(result$estimate), t_statistic =
result$statistic,
             p_value = t_test_bonferroni[i], conf_low = result$conf.int[1], conf_high =
result$conf.int[2])
}))
print(results_df)

# Demographic Comparisons

# Ensure the grouping variable is a factor
demographics$group <- as.factor(demographics$group)

# List of Kruskal-Wallis test variables
kruskal_vars <- c("Age_at_Assessment", "Age_at_Diagnosis", "Time_Since_Diagnosis",
"EDSS")

# Perform Kruskal-Wallis tests and store p-values
kruskal_results <- lapply(kruskal_vars, function(var) {
  kruskal_test <- kruskal.test(as.formula(paste(var, "~ group")), data = demographics)
  kruskal_test$p.value
})

# Apply Bonferroni correction
kruskal_bonferroni <- p.adjust(kruskal_results, method = "bonferroni", n =
length(kruskal_results))

# Print results
cat("Kruskal-Wallis Test Results (Bonferroni Corrected):\n")
for (i in seq_along(kruskal_vars)) {
  cat(kruskal_vars[i], ": p-value =", kruskal_bonferroni[i], "\n")
}
```

```
# Perform chi-squared tests for each variable

# List of chi-squared test variables
chi_vars <- c("Sex", "Number_Clinical_Events", "Number_Optic_Neuritis_Events")

# Perform chi-squared tests and store p-values
chi_results <- lapply(chi_vars, function(var) {
  chi_test <- chisq.test(table(demographics[[var]], demographics$group))
  chi_test$p.value
})

# Apply Bonferroni correction
chi_bonferroni <- p.adjust(chi_results, method = "bonferroni", n = length(chi_results))

# Print results
cat("Chi-Squared Test Results (Bonferroni Corrected):\n")
for (i in seq_along(chi_vars)) {
  cat(chi_vars[i], ": p-value =", chi_bonferroni[i], "\n")
}

# PLS Path Modeling
my_PLSPathModel_data <- read_excel("AlDahhanetal_BrainComm_pathmodel.xlsx")

# Define path matrix (inner model)
PLS_path <- rbind(
  c(0,0,0,0,0,0),
  c(1,0,0,0,0,0),
  c(1,1,0,0,0,0),
  c(1,1,0,0,0,0),
  c(0,1,1,0,0,0),
  c(0,1,0,1,0,0)
)
colnames(PLS_path) <- rownames(PLS_path)

# Define blocks of indicators (outer model) and modes
PLS_blocks <- list(4:9, 10:305, 369:431, 306:368, 304, 305)
PLS_modes <- c("A", "A", "A", "A", "A", "A")

# Run PLS path analysis
PLSModel_f <- plspm(my_PLSPathModel_data, PLS_path, PLS_blocks, modes = PLS_modes)

# Plot barchart of loadings
ggplot(data = PLSModel_f$outer_model, aes(x = name, y = loading, fill = block)) +
  geom_bar(stat = 'identity', position = 'dodge') +
  geom_hline(yintercept = 0.7, color = 'gray50') +
  ggtitle("Barchart of Loadings") +
  theme(axis.text.x = element_text(angle = 90))
```

```
# Print summarized results
summary(PLSModel_f)
plot(PLSModel_f)

# Bootstrap for PLS Path Modeling
PLSModel_bootstrap <- plspm(my_PLSPathModel_data, PLS_path, PLS_blocks, modes =
PLS_modes, boot.val = TRUE, br = 5000)

# Display bootstrapped results
print(PLSModel_bootstrap$boot)
print(PLSModel_bootstrap$effects)
```
